# Experiences of patients with complex needs at municipal emergency outpost satellites

**DOI:** 10.1080/02813432.2025.2502095

**Published:** 2025-05-09

**Authors:** Trude Østerbø, Gro Hovland, Siri Ytrehus, Dagrun Kyrkjebø, Erik Zakariassen, Ole. T. Kleiven

**Affiliations:** a Western Norway University of Applied Science, Førde, Norway; b National Centre for Emergency Primary Health Care, NORCE, Norwegian Research Centre, Bergen, Norway; c Department of Global Public Health and Primary Care, University of Bergen, Bergen, Norway

**Keywords:** Video conferences, emergency outpost satellites, complex needs, patient experiences, health communication

## Abstract

**Background:**

In 2015, Norway introduced stricter requirements for organizing primary emergency care. These changes led to new solutions such as municipal emergency outpost satellites, providing inhabitants shorter access to care in resource-limited areas. This study explores patient experiences at emergency outpost satellites staffed by a nurse on site with a general practitioner (GP) available *via* video consultation.

**Methods:**

A qualitative study was conducted with seven patients, aged 62–82, with complex needs, from four small and medium-sized municipalities in Vestland County, Norway. Data were collected through individual semi-structured interviews, which took place within 6 months of an acute consultation at an emergency outpost satellite. The data were analysed using systematic text condensation (STC).

**Results:**

Patients had both positive and negative experiences with video consultations. They valued short travel distances and quick GP access *via* video link. Video consultations often replicated several aspects of in-person visits, with nurses playing a crucial role in organizing and ensuring that care was provided effectively. Nurses were key mediators, supporting patients before, during, and after the consultation. However, some patients were dissatisfied with the lack of a physical GP presence, technical issues, and communication challenges.

**Conclusions:**

The participants’ experience of communication with the GP and nurse was crucial for the video consultation to be perceived as satisfactory. Participants felt that video consultations reduced travel burdens and provided quick clarification. Nurses played an important mediating role, but poor communication and technical issues made some participants feel unsafe using the emergency outpost satellites.

## KEY POINTS

**Access to Care:**

Patients valued the shorter travel distances and immediate access to a GP *via* video consultations.

**Nurses’ Roles:**

On-site nurses were seen as key intermediaries, supporting communication between patients and the GP.

**Patient Experiences and Barriers:**

While some patients reported positive experiences with video consultations replicating in-person visits, others expressed dissatisfaction with the lack of a physical GP presence, technical issues, and communication challenges, which impacted the effectiveness and overall patient experience.

## Background

In 2015, Norway introduced stricter requirements for organizing primary emergency care [1]. This led to the development of new solutions such as municipal emergency outpost satellites. These satellites were designed to provide inhabitants in resource-limited areas with more rapid access to care [2]. Municipalities in Norway are responsible, together with the state-owned hospital Health Trusts, for ensuring that their population can access adequate emergency medical help when needed [1]. The municipalities are responsible for out-of-hours, urgent, and emergency primary care with a general practitioner (GP) on call, while the Health Trusts are responsible for secondary emergency care, with the ambulance service serving as prehospital resources. The GP on call is expected to do call-outs with the ambulances in acute patient situations. However, due to a heavy workload and other factors, many rural municipalities have found it difficult to recruit and retain nurses and doctors with sufficient emergency medical competence [3–5]. As a result, municipalities are increasingly offering their primary emergency medical services through inter-municipal consortiums [2], which has led to patients in many areas now having longer distances to the primary emergency services (casualty clinic) [6].

The Coordination Reform (2012) and changes in the Emergency Medicine Regulations in 2015 laid the foundation for strengthening municipalities’ emergency medical services, which led to the establishment of new models such as emergency outpost satellites [1,7,8]. In 2019, the Directorate of Health established the model of an emergency outpost satellite staffed with a nurse who had access to a GP *via* video link. Emergency outpost satellites were established in five municipalities with a total population of 13,000 inhabitants. These municipalities, with populations ranging from 1,000 to 4,500, are part of an inter-municipal consortium within the emergency primary care out-of-hours service. All the inhabitants in the municipalities with emergency outpost satellites are more than a 1hour drive from the inter-municipal casualty clinic. All patients have to first call the Local Emergency Medical Communication Centre (LEMC) for assessment. Patients without critical conditions but needing treatment that cannot wait until the next day are referred to the satellite outposts. There, the patient meets a nurse who conducts the preliminary examination, while further examination and treatment are carried out in consultation with a GP from the LEMC *via* video [2].

The goal of the emergency outpost satellites is to ensure an adequate emergency medical service for the residents of areas far from the casualty clinic, while also strengthening and preserving the competence already in the municipalities [4,5]. Further objectives have been to improve patient-perceived quality in rural and remote areas, reduce transport costs, and make better use of standby resources in the municipality [2].

Previous research has shown that telemedicine during planned consultations can reduce costs, spare patients a long journey, and effectively facilitate referral and treatment of patients in remote areas [9–13]. Studies of patients’ experiences with the use of video consultations during planned consultations show that they are considered a good alternative to an in-person consultation in situations where a physical examination is not necessary [9,14]. Video consultations can reduce the number of patients who must travel to the hospital but can be perceived as less safe due to technical challenges and the risk of misdiagnosis [15]. The quality of video consultations in emergency care is highly dependent on the nurse’s ability to act as the hub during the consultation, and the GPs participating *via* video play an important role in maintaining the patient’s sense of safety and inclusion [16].

Little of the literature on video consultations pertains to the context of emergency primary care services [17]. Some studies show that video consultations with a doctor in emergency situations can facilitate the start of necessary medical treatment in rural and remote areas and quickly call out an ambulance if deemed necessary [10,18]. Emergency nurses in ambulances found that video consultations created safety and involved the patient in the treatment situation because the doctor participated in video decisions [19,20]. Increased insight into patients’ experiences at emergency outpost satellites can generate new knowledge about the use of video consultations and the GP’s role in supporting both the nurse and the patient during video consultations.

Elderly patients with complex and multimorbid chronic conditions face increased medical challenges and often require more frequent visits to emergency departments [20,21]. Older adults with complex health needs require a holistic and coordinated approach to healthcare, with primary healthcare playing a central role, while specialized services are available when needed [22]. Clinical guidelines are developed for individual diagnoses, while patients with a composite disease picture experience that the health service is fragmented and poorly coordinated in relation to their needs for support [23]. Nurse-led emergency outpost satellites, with a GP available *via* video link, are a new service in remote small- and medium-sized municipalities. The participants in this study have complex health needs, and while the service may offer quicker support, it remains unclear if it fully meets their needs. Exploring patient experiences of this service is therefore of interest. Given the challenges of providing timely and high-quality healthcare to patients in remote areas, understanding these experiences is essential for improving emergency medical services in rural settings. This study investigated patients’ experiences with emergency outpost satellites in terms of their safety and the quality of medical treatment. The aim of the study was to explore how elderly patients with complex health needs experience consultations with a GP *via* a video link at a local emergency outpost satellite manned by a nurse. This exploration was guided by the following research questions:

### Research questions

- How did patients experience waiting time to meet the GP on video, treatment time, availability, facilities, and equipment?
- How did patients experience the meeting and communication with the nurse and GP before and during the video consultation?
- How did patients experience the nurse’s competence to understand their health problems during the consultation?

## Methods

### Study setting

Patients from four small and medium-sized municipalities in Vestland, a county in Norway, participated in the study. The four emergency outpost satellites, located at the nursing home in each municipality, are open out of hours. They are equipped like the regular inter-municipal casualty clinic within the primary emergency out-of-hours services; that is, they have the necessary equipment and examination rooms. Before the launch of the emergency outpost satellites, all nurses and GPs received joint training to operate the outposts in the municipalities. The nurses completed a course in emergency medicine, where they received training in triaging, and undertook internships at the inter-municipal casualty clinic, where they gained practical experience. This training was crucial to ensure that the healthcare staff had the necessary competence to manage patients with complex health needs at the outpost satellites. The nurses were also trained in video consultations and holistic patient care in rural areas. The GPs were trained in video consultations and given information about equipment, medications, and nurse competencies at each outpost satellite. Monthly simulations were held with the satellite nurse, LEMC, and the on-call GP. The inter-municipal casualty clinic is in the same building as the city’s hospital. The outpost satellites are each staffed at any time with one nurse from a roster of nurses who also work in the regular primary care service in the municipality [2].

Patients must call the LEMC as usual, where nurses triage the situation and determine if the patient should be referred to the inter-municipal casualty clinic or an emergency outpost satellite. Calls triaged as red responses (highest urgency) are immediately transferred to the Emergency Medical Communication Centre (EMCC; dispatch centre). The decision regarding where the patient should be treated is based on medical information from the caller in combination with the use of a decision support tool (the severity and urgency of the situation), factors such as the distance to the casualty clinic and the availability of ambulance services, and other relevant considerations [2]. The nurse at the emergency outpost satellites performs examinations, observations, and interventions in cooperation with the GP on call at the inter-municipal casualty clinic, who conducts a video consultation with the patient. In addition to treating patients at the emergency outpost satellites, nurses in one municipality, with no ambulance nearby, can also respond to emergencies at the patient’s location. In the event of a doctor–ambulance alarm from the EMCC (red response), the satellite nurse provides initial care until the nearest ambulance arrives [2].

## Design

To explore patients’ experiences and perceptions of consultations at municipal emergency outpost satellites, we employed a qualitative approach using systematic text condensation (STC) to analyse semi-structured interviews [24].

### Inclusion criteria

Patients who had been to a consultation at a municipal emergency outpost satellite and met the following inclusion criteria:

- aged between 60 and 90 years
- diagnosed with two or more chronic diseases
- competent to consent and able to understand spoken and written Norwegian
- time between the consultation and our interview did not exceed 6 months

### Recruitment process

Patients who received care at emergency outpost satellites for acute illness between 1 September 2020 and 28 February 2021 and met the inclusion criteria were contacted by a GP from the inter-municipal casualty clinic.

The GP began recruitment by reviewing the appointment list to identify patients marked as satellite patients. All individuals aged 60-90 years were selected. Patient records were checked for comorbidities, and their survival status was verified. Capacity for consent was assessed by reviewing records, and in some cases, the GP consulted with other healthcare professionals. When patients were deemed capable, they were contacted by phone, provided with both verbal and written information about the study, and asked if they would consent to an interview. In certain cases, home care staff were present during the call to facilitate communication and ensure accurate information transfer. The process ensured that all patients could provide informed consent following Norwegian legislation [25].

Between 1 September 2020 and 28 February 2021, there were a total of 7,707 contacts to the LEMC. Of these, 1,982 individuals were between 60- and 90-years-old. During the same period, a total of 49 patients visited the emergency outpost satellites. A total of eight patients met the inclusion criteria and were contacted, of whom seven agreed to participate in this study. After providing written consent, the participants were contacted by the first author to arrange an interview. Interviews were conducted continuously as participants provided written consent. To ensure that the participants remembered details of their consultation, a requirement was set that no more than 6 months had passed between the consultation and the interview. The participants lived at home. Due to the COVID-19 pandemic, informants were offered the option to participate in interviews in person, by phone, or *via* Microsoft Teams.

### Demographics

The sample consists of five women and two men aged 62 to 82 years from four of the region’s five municipalities that have an emergency outpost satellite. The patients in the study had two or more chronic diseases, about which the study did not collect information.

### Ethical considerations

The study has been approved by the regional committees for medical and health research ethics (REK West reg. no.171692) and reported to the Norwegian Centre for Research Data (NSD reg. no. 925342). All informants were informed of their rights and signed written consent forms before the interviews. Signed consent forms were uploaded to the Western Norway University of Applied Sciences’ research server before data collection. Patients’ data have been anonymized.

### Data collection

The interviews were conducted consecutively after approvals and consent were granted, within a time frame of 6 months following consultation at the municipal emergency outpost satellite. Six patients were interviewed within 2-3 months after the consultation, and the seventh patient approximately after four months. The interviews were originally collected as part of a master’s thesis project. Seven individual interviews were conducted, all carried out by the first author using a semi-structured interview guide that was adjusted after two pilot interviews. Following the pilot interviews, an open question about why the patient had visited the emergency outpost satellite was included. The interview guide was developed based on themes related to the research questions, which included four main topics: technical equipment and availability; communication and collaboration; safety and competence; and quality. The interviews began with an open question about why the patient had visited the emergency outpost satellite, and follow-up questions were selected based on the responses provided by the informants [24]. The interviews lasted between 15 and 40 min, and at the end, informants were asked if they had anything further they wanted to add. Three interviews were conducted face-to-face, while four were conducted by phone; none of the informants chose to participate *via* video interview. The fact that only three informants opted for in-person meetings can likely be attributed to the ongoing COVID-19 pandemic. Audio recordings were made and transcribed verbatim by the first author after each interview; the first author also wrote field notes capturing their impressions and descriptions immediately after each interview. The first author contacted one informant after the interview to confirm that information had been correctly understood. The names of the informants and municipalities were coded and anonymized [24]. Quotes in the results section are coded by municipality [1–4] and patients are numbered [1–7].

### Reflexivity

The first author, a nurse with work experience in emergency care at a municipal outpost satellite prior to the study, has no professional relationship with the municipalities included in the study. Throughout the research process, the first author has been aware of their preconceptions and how these might influence the study, particularly given their direct experience in the field. The first author’s professional background provided a deeper understanding of the emergency outposts’ organization, equipment, and practical aspects, facilitating follow-up questions during interviews. However, we acknowledge that this background may have influenced the research, aligning with Malterud’s [24] view on reflexivity, where the researcher’s experiences and preconceptions can shape the interpretations. Reflexivity has been an integral part of the research approach, ensuring critical assessment of whether this professional background could shape the research process and findings.

### Analysis

The data material was analysed with STC [24]. The first author transcribed the interviews verbatim, and the project group conducted analyses. This article is based on a reanalysis of interviews originally collected as part of a master’s thesis project [26].

**Figure 1.**
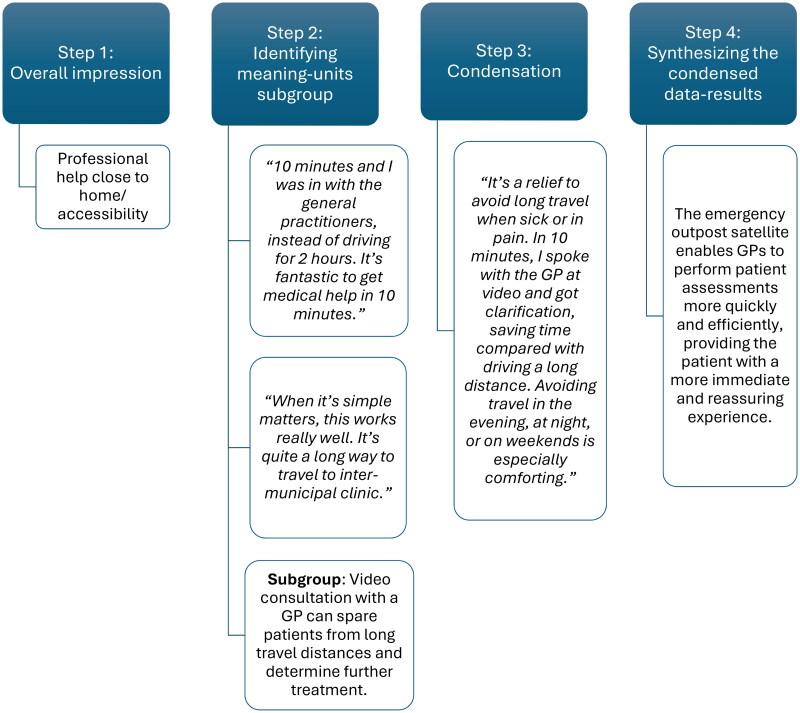
Provides an example of the analysis steps for one theme.

The preliminary themes, developed during the original master’s thesis analysis, served as a foundation for the reanalysis process. In the first step of STC [24], the data material was read in full to obtain an overall impression, and preliminary themes were identified. These themes were then revisited and refined to guide the subsequent analytical steps. In the second step, meaning units relevant to the preliminary themes were identified across the data material. TØ, GH and OTK collaborated to extract these units and organize them into code groups. All changes and decisions throughout this process were carefully documented by the first author, ensuring transparency and traceability. In the third step, the content of each code group was systematically condensed. This involved summarizing the meaning units into condensates that captured the essence of participants’ experiences and perspectives, organized within each code group to uncover more nuanced patterns. In the fourth and final step, the condensed meaning units were synthesized into analytical text, resulting in four main categories that reflected the central themes of the data [24]. The draft analysis was reviewed by the remaining project team members (SY, EZ, and DK), who contributed to further refinement of the categories to ensure analytical depth and coherence [24] (Figure 1). The second step presents a draft of the meaning-units that have been organized into subgroups.

## Results

This study explored patients’ experiences with video consultations at emergency and casualty services in rural areas. The analysis was broken down into four main categories, which are presented in the results sections below. While patients appreciated being assessed locally, some felt that video consultations were insufficient for more serious conditions and preferred in-person consultations. The nurse’s role was considered crucial, with patients expressing trust in their competence and support during the consultation. Several patients reported positive experiences, though some encountered communication challenges and technical issues. The nurses’ proficiency with the technical equipment was important in minimizing these challenges. Patients felt secure when the nurse initiated the diagnostic process and provided support throughout the consultation.

### Patient experiences with access to local emergency care: advantages and limitations of video consultations at an outpost satellite

All patients agreed it was an advantage to travel only a short distance when they were sick and in pain. Patients were assessed at the outpost satellite during evenings, weekends, and holidays. They appreciatively noted the short travel time and a shorter waiting time compared with previous experiences at the inter-municipal casualty clinic. Sometimes a patient needed to be hospitalized, while other times the patient was treated at the outpost satellite. In one patient situation at the outpost satellite, the GP’s assessment *via* video was crucial, as the situation was more serious than was initially thought. In this situation, the GP on call called a physician from the specialist health service to assess the injury *via* video. The patient describes the cooperation between the municipality and the specialist health service as very good in this situation. Several patients, noting the possibility of a quick clarification by the GP *via* video consultation, described the outpost satellite as a safe local emergency offer.

10 minutes and I was in with the general practitioners, instead of driving for 2 hours. It’s fantastic to get medical help in 10 minutes. (Patient 4.3)

Some patients felt that their condition was too serious to be assessed at the outpost satellite. These patients were examined at the outpost satellite, and the GP on call assessed that they should be followed up by their own regular GP. In retrospect, they thought a lot about what had happened during the video consultation, and they expressed dissatisfaction with the diagnosis and treatment. These patients opined that video consultations work when simple cases need to be assessed and treated but that in their situation it would have been better to go directly to the emergency primary care centre for an in-person consultation with the GP on call.

In retrospect, I don’t know if it would have been better to have gone to the hospital, but since you don’t get the offer either, then you’re kind of cut off from it. You’d like to get an examination as soon as possible…. (Patient 2.2)

### Nurses’ expertise is crucial to patients’ trust in emergency outpost satellites

Patients expressed trust in the nurses’ competence. Nurses came out to meet the patients outside the municipal outpost satellites and accompanied them throughout the consultation. Several patients in the sample appreciated that they already knew the nurses from before, which made them feel safe.

She was very clear. I spoke with her directly; I was transferred to her. She was very polite, organized, kind, and skilled. I know her well, and she is an experienced nurse, which was very reassuring. (Patient 6.4)

Patients expressed that the nurses were quick and took various tests and measurements to start the diagnostic process. After a patient called the LEMC about an irregular heartbeat, the satellite nurse came to the patient’s home very quickly and then sent electrocardiogram images and other necessary measurements to the GP for assessment. The patient felt that the satellite nurse conducted a thorough examination. The patient reported that she was no more anxious here at home than if she had gone directly to the hospital.

If it really is urgent, like a stroke or a heart attack, then help comes quite quickly, and they order a helicopter if you need it. If not, they send you in an ambulance – you don’t lie at home alone for many hours, waiting for something to happen. (Patient 5.3)

### A sense of safety emerges in video consultations when the nurse and GP work seamlessly together and involve the patient effectively

Patients said that video consultations with a GP worked well. They reported that there had been good dialogue, and they had been able to ask questions of the doctor and see a clear image of the GP on the screen. The GP was in this way experienced as ‘present’ in the room. It was important to one patient that he had made eye contact and remembered the GP’s face afterwards.

The image remained consistently sharp throughout the session, which might require some technical training, but I found it perfectly adequate. The experience felt very professional and engaging. The GP was fully present, and I felt a strong sense of eye contact with him. (Patient 6.4)

Many patients stated that they felt positive about the new technology. They agreed that it was different compared with a face-to-face consultation with a GP, but that it is a development that will become more common in the future. Patients expressed that the video consultation went well since the doctor was helpful and skilled, and the nurse could do the necessary examinations. Patients positively evaluated the cooperation they had witnessed between nurse and doctor: the nurse took the medical history, and the doctor assessed what should happen next. It was safe to have a nurse there during the consultation and while waiting for further help.

I felt they were consistently proactive, striving to find solutions as soon as possible, providing reassurance that they were in control and that I would receive further assistance if needed. There was good collaboration between the doctor and nurse, in my opinion. (Patient 4.3)

Some patients described communication challenges during the video consultation, for example, that the GP did not speak directly to them but mostly turned to the nurse. Patients were also waiting a while before the GP came on the screen. One patient, who described the video call as very short, said the GP had told them early on that the casualty clinic in Førde was busy and discouraged them from going there. Another patient reflected that she had been passive because of her illness and gave this as an explanation of why she had not developed a good relationship with the doctor during the video call.

Yes, but she mostly directed her attention to the nurse. She asked me one or two questions directly, but, overall, there wasn’t much dialogue. I believe we would have had a better conversation if we had met in person; she wouldn’t have seemed so busy. (Patient 7.4)

### Technical difficulties with the equipment may detract from patient confidence in video consultations

Some patients reported that the doctor came quickly on the screen, and they praised the nurses’ competence with the technical equipment during the consultation. Several other patients reported that they had had to wait for the doctor to come on the screen and said the nurse had struggled with the videoconference equipment. Most patients said that the nurse’s interaction with the video equipment itself had little effect on the consultation.

Yes, once they got it up and running, we had to wait for the general practitioners to become available. I remember that. (Patient 7.4)

One patient reported that problems with zooming in to make an injury visible on the screen had left them unsure whether the assessment was correct. The patient mentioned that a function to see oneself on the screen could be advantageous.

It felt a bit like ‘waving to the audience’ (laughs). I felt a bit silly in that moment, with a huge screen in front of me. It would have been nice to know if he could actually see my hand, or if it was more like a royal wave. (Patient 6.4)

## Discussion

Video consultations can reduce the number of patients sent to the casualty clinic and save patients a long travel distance. However, outpost satellites can be perceived as unsafe due to the lack of physical presence of a GP, technical challenges, and communication issues before and during the video consultation. The nurse plays a central and important role both before and during the video consultation by meeting patients outside the municipal outposts satellite, accompanying them inside, providing information, and initiating the diagnostic process.

### Communication with the GP in the video consultations

Previous studies of in-person and remote healthcare encounters have shown that patients evaluate the quality of service based on their perception of the healthcare professionals’ knowledge, communication within the clinical relationship, and degree of satisfaction of the patients’ needs and desires [27–31]. Most patients in this study reported positive experiences of the actual meeting at the municipal outpost satellite *via* a video link with the GP located at the inter-municipal casualty clinic. They described good dialogue and a clear image of the doctor on the screen. Such patients believed that, though a video consultation could not be compared with a physical meeting with a GP, it was a good alternative.

Some patients expressed that they felt unseen or unheard by the GP during the video consultation: for example, the doctor was busy and mostly directed their attention to the nurse at the satellite post. Some patients stated that they were not taken seriously and believed that the treatment would have been different in a physical consultation at the inter-municipal clinic in Førde or at the hospital. The patient’s vulnerability and lack of involvement in the video consultation can lead to uncertainty about the GP’s assessment of the situation and whether the diagnosis and treatment are correct. This is supported by previous research [27,32,33]. Adequate training for GPs and nurses is essential to ensure the quality of video consultations. When inter-municipal satellite clinics were established, training sessions that brought GPs and nurses together gave them an opportunity to better understand each other’s roles and competencies. Video consultations also require sufficient staffing at the inter-municipal casualty clinic, with at least two GPs present on duty [2].

In our study, patients’ descriptions of communication with the GP during the video consultation at the municipal outpost satellites were consistent with earlier findings. Studies show that patients communicate less about their issues during video consultations than in physical consultations, and that healthcare professionals also provide less information during video consultations than in face-to-face meetings [34]. Lack of information about the situation can lead to a significant difference between the patient’s assessment of their health condition and the healthcare professional’s evaluation [28,32,35]. These findings are also consistent with studies on GPs’ experiences with video consultations. These highlight that, although video consultation can be practical, informative, and effective, particularly when the GP knows the patient, communication during video consultation tends to follow a limited style and a ‘one-issue approach’, and the video format diminishes the nonverbal cues that are more prominent in in-person consultations. Over time, the frequent use of video consultations may undermine the establishment and maintenance of relational trust, which could negatively impact the quality of care and patient safety [34].

### Technological challenges in video consultations

Digitalization in the healthcare sector creates new roles and requires new competence. If the nurse has received training in and has good knowledge of the technical equipment, the new technology can be used to focus on person-centred care and communication [29,35]. In our study, patients’ perceptions of the nurses’ competence with the technical equipment differed from one municipality to another, possibly because of different training and less depth of experience with the tools at some sites. The regulations require municipalities to ensure that outpost satellites have the necessary equipment available for diagnosis, treatment, and the right competence to handle it [1].

Several patients in our study reported that the nurses struggled to establish a video connection at the emergency outpost satellite. As described above, one patient had doubts about the GP’s assessment as a result. Our informants are older patients, who may be more sceptical than younger peers about the use of technological solutions to replace a physical meeting [30]. Previous studies show that patients can experience video consultations as unsafe as a result of technical challenges [14,31,36]. Patient experiences from another study assess that diagnostic resources at local facilities are effective in some situations, while in more serious patient situations, the diagnostic resources at outpost satellites are seen as inadequate compared with the resources at the hospital [36,37]. This corresponds with the results of this study, which show that perceived uncertainty in diagnostics makes patients unsure whether the assessment is correct, and that not all medical conditions are suitable to be assessed at an emergency outpost satellite.

### The local emergency primary care services

Patients want functioning emergency primary care services to be offered locally [38–40]. This corresponds with the patients in our study who were satisfied with getting treatment at the local outpost satellite, near home. These patients experienced shorter waiting times than at the inter-municipal clinic. A quick clarification by the GP was singled out as crucial for what should happen next. In one patient situation, the emergency doctor called a doctor from the specialist health service, and they assessed the patient together *via* video. Previous research indicates that the use of telemedicine in prehospital services can provide essential emergency medical treatment to patients in remote areas when a doctor is not available on site [9,15,40]. Additionally, it can serve as a guiding resource when the hospital is far away [18,22].

All patients in this study described travel distance as an additional burden when one is sick and in pain. Other studies show that travel distance is a decisive factor for how many use the emergency room: in cases with either low or high urgency, the further patients must travel to the emergency room, the less likely they are to use it [6].

### Nurses’ mediating role

Patients described the nurses as competent, helpful, and accommodating both before and during the video consultation with the GP. Several patients knew the nurses, whose primary workplace was either in nursing homes or home care services in the municipality [2], from these other services and from previous consultations at the outpost satellite, which made them feel safe. When a nurse is familiar with the complex needs and medical history of patients with several chronic diseases, such as those enrolled in this study, this is important for patient safety and reassurance [27,30,33,34].

According to most patients, the nurses created a safe atmosphere at the outpost satellite by making necessary observations and starting the diagnostic process early. The nurse’s competence in emergencies was described as reassuring and important, considering that the GP was several hours away. The nurses at the emergency outpost satellite have completed emergency medical courses. Previous research points out that the nurse’s competence and knowledge of the patient’s condition contribute to better communication with the GP [41]. This is an effective use of the nurses’ competence and also helps meet the residents’ right to emergency health care where they live [1,3].

Several patients describe good cooperation between the nurse and the GP. The nurse performed necessary examinations while there was close interaction with the GP *via* video. Other studies from prehospital services confirm that video consultations can strengthen patient safety, as the GP can see the patient and make medical assessments together with the nurse [19,20,42].

Several patients argue that outpost satellites are suitable only when there are simple issues to resolve; in situations that patients perceive as serious, they prefer a physical meeting with the GP. This confirms findings in other studies [14,36,43]. Patients with multiple complex conditions pose an additional challenge for the nurse and GP at the urgent care clinic. In situations where the GP clarifies the situation and determines that it is not urgent, the patient is advised to contact their regular GP during the daytime for further follow-up. This is consistent with central guidelines and legislation [1,2]. In light of this, there may be a different perception of what situations patients assess as urgent, and of the doctor and nurse’s intentions to assess and treat the degree of illness [44].

### Strengths and weaknesses of the study

Informants were recruited by a GP at the inter-municipal casualty clinic, after a collaboration meeting where the inclusion criteria were discussed. This made the researchers neutral in the selection. Recruitment took place during the COVID-19 pandemic. The outpost satellite clinics were closed to patients with respiratory symptoms, likely decreasing the overall patient flow to the clinic and the recruitment base for the study. To ensure information power [45] and a suitable sample that met the inclusion criteria, these criteria were carefully reviewed with the person responsible for recruitment. This process helped to include participants with relevant experience and insights that could address the research question. After six interviews, we assessed that the data were sufficient to answer the research question, and a seventh participant was included to further strengthen data collection. At this point, we considered the data to be detailed and informative enough to provide valuable insights [24,45] and we proceeded with data analysis. In line with Malterud et al. [45], information power depends on how well the sample can contribute relevant information, rather than the number of participants. The research question played a key role in ensuring that the informants had the necessary experience and insights to address the topics thoroughly. This step helped strengthen the validity and depth of the collected data.

Two phone interviews lasted 15–25 min, compared with up to 40 min for face-to-face interviews, which can be attributed to the more direct nature of phone interviews, often leading to shorter conversations. Research suggests that phone interviews are typically shorter than face-to-face interviews, with participants less likely to engage in small talk. This may have made the interviews more efficient, but it can limit some depth of depictions and descriptions as in a face-to-face interview [46].

In this study, each interview was conducted within 6 months after the corresponding consultation. Six patients were interviewed within 2-3 months after the consultation, while for the seventh patient, approximately four months had passed. However, all interviews were conducted as soon as the patients had signed the consent form. Long intervals between events and interviews may affect participants’ ability to recall details accurately, potentially leading to inconsistencies in their narratives. External factors such as personal circumstances or environmental conditions may change, influencing participants’ perspectives and responses. Long intervals can also lead to challenges in data analysis, because evolving contexts can complicate the interpretation. Most of the interviews were conducted within a relatively short period of 2-3 months, and as the informants provided rich and detailed accounts of their consultation experiences, we concluded that these potential limitations did not significantly impact our findings.

The small sample size was balanced by the richness and quality of the data, which strengthened its internal validity. While the findings cannot be generalized to a larger population, the limited number of participants provided detailed insights. The study’s focus on patients’ experiences with video consultations resulted in valuable, context-specific findings that could contribute to improving video consultation concepts. In terms of external validity, the results may be relevant to other healthcare settings using video consultations, particularly in similar contexts. Despite the small sample size, the study demonstrated sufficient information power, as described by Malterud et al. [24,45].

During the interviews, open questions were asked, and informants were encouraged to speak freely about their experiences. Follow-up questions were asked based on what was said [24] and to cover all topics in the interview guide. The first author, who conducted the interviews, has experience in patient-centred emergency care and general practice. This background made it easier to ask follow-up questions, particularly regarding the organization of the emergency outpost, allowing for a deeper exploration of how patients experienced this aspect of the service. At the same time, there was a constant awareness of potential preconceptions and how these might influence the study design and data interpretation. For example, the first author had the assumption that older patients might have a negative attitude toward video consultations and feel uncomfortable being examined by a nurse instead of a GP. While this experience and background may have deepened the understanding of the topic, it also carried a risk of bias in interpreting the data.

However, the diverse backgrounds within the research team strengthen the validity of the findings, because the team actively worked to challenge individual biases throughout the process, according to Malterud [24].

### Implications for practice and further research

The findings in this study indicate that the emergency outpost satellites require competence on the part of the nurse and doctor, cooperation between them, good training on the technical equipment, and clear communication between the patient and doctor during the video call. More research is needed on video consultations in the context of emergency services. Further study could also clarify, for example, which situations are too serious to be suitable for assessment *via* video.

## Conclusions

The Participants’ experience of communication with the GP and nurse is an important starting point if video consultation is to be experienced as satisfactory. The participants here experienced that video consultation with a GP saved them from burdensome travel and provided a quick clarification of their situation. Nurses played a central mediating role, providing patients with information about the assessments. However, perceived poor communication and technical problems during video consultations made some respondents feel unsafe using emergency outpost satellites.

## Supplementary Material

Interview Guide_revision 2.pdf
