# Supplementary material for: Experiences of patients with complex needs at municipal emergency outpost satellites
Source: Scand J Prim Health Care. 2025 May 9;43(4):733–44. doi: 10.1080/02813432.2025.2502095 (PMC12632231; doi:10.1080/02813432.2025.2502095)
Supplement: Interview Guide_revision 2.pdf [file IPRI_A_2502095_SM1340.pdf]

Experiences of patients with complex needs at municipal emergency outpost satellites

INTERVIEW GUIDE

Semi-structured individual interview of about 1 hour. The interview will be recorded.

Introduction

Would you like to say something about the reason you contacted the Local Emergency Medical Communication Centre (LEMC)?

Technical equipment and accessibility

How did you experience the waiting time, treatment time, accessibility, premises, and equipment?

- Local treatment versus travel?

Communication and collaboration

How were you met by the nurse during the consultation?

How did you experience the video consultation?

- How did you experience the communication between you and the nurse/doctor?
- What are your thoughts about the doctor not being physically present?
- Were you prepared for the doctor to only be available via video consultation?

How was the collaboration between the doctor and the nurse?

- How were you heard and included in the treatment situation?
- How was this consultation different from other consultations?

Safety and competence

How did you experience the nurse's competence during the consultation?

- How were you cared for considering this was an urgent consultation?
- To what extent did you feel that you received the help you needed?
- What knowledge did the nurse have about the fact that you have multiple diagnoses?

Quality

How did you experience the quality of the service?

- Was it positive? Negative? Examples?
- In what way could something have been done differently?
- What do you think about this being a service that could be relevant in the coming years?

Thank you for participating in the interview, is there anything else you would like to add?
